# Chronic corticosterone administration in adolescence enhances dorsolateral striatum-dependent learning in adulthood

**DOI:** 10.3389/fnbeh.2022.970304

**Published:** 2022-08-12

**Authors:** Ty M. Gadberry, Jarid Goodman, Mark G. Packard

**Affiliations:** ^1^Department of Psychological and Brain Sciences, Texas A&M University, College Station, TX, United States; ^2^Department of Psychology, Delaware State University, Dover, DE, United States; ^3^Department of Psychological and Brain Sciences, Texas A&M Institute for Neuroscience, Texas A&M University, College Station, TX, United States

**Keywords:** early life stress (ELS), memory, habits, learning, striatum, corticosterone

## Abstract

Previous evidence indicates a link between early life stress (ELS) in humans and a predisposition to psychopathologies that are characterized in part by maladaptive habitual behaviors. Stress and anxiety influence the relative use of mammalian memory systems implicated in these disorders. Specifically, cognitive memory functions of the hippocampus are typically impaired by stress/anxiety, whereas habit memory functions of the dorsolateral striatum (DLS) are enhanced. A stress/anxiety bias toward habit memory has largely been demonstrated in adult rodents and humans, and the effects of ELS on the later use of DLS-dependent habit memory in adult rodents have not been extensively examined. The present study addressed this question by chronically elevating corticosterone (CORT) during adolescence, and investigated the effects of this treatment on DLS-dependent habit learning in adulthood. In experiment 1, adolescent rats received a single daily injection of either CORT (5 mg/kg) or vehicle (cVEH) over 5 days and then matured undisturbed before training as adults in a DLS-dependent water plus-maze task. Rats administered CORT injections during adolescence displayed a strong trend toward enhanced learning during adulthood relative to vehicle-treated rats. Adolescent CORT administration also increased anxiety-like behavior in adulthood in an elevated plus-maze. In experiment 2, adolescent CORT administration enhanced task acquisition in adulthood, and this effect was blocked by concurrent administration of the glucocorticoid antagonist mifepristone (30 mg/kg). Taken together, these findings suggest that chronic elevation of glucocorticoids during adolescence are sufficient to facilitate habit learning in adulthood, and indicate that glucocorticoid function may be a potential underlying mechanism by which ELS influences subsequent habitual behaviors.

## Introduction

Emotional arousal including stress and anxiety has a dramatic effect on multiple memory systems of the mammalian brain, and evidence has indicated both enhancing and impairing effects. One of the factors that determine whether emotional arousal enhances or impairs memory is the memory system being investigated (for reviews see Packard and Goodman, 2012, 2013; Schwabe, 2013; Packard et al., 2018). Specifically, previous research has indicated that stress and anxiety typically impair hippocampus-dependent cognitive memory, while enhancing dorsolateral striatum (DLS)-dependent habit memory. These effects of emotional arousal on multiple memory systems have been demonstrated in both rodents and humans. For example, in rats, several methods of inducing robust emotional arousal, including restraint stress and foot shock (Kim et al., 2001), anxiogenic drug administration (Packard and Wingard, 2004; Leong et al., 2012), and exposure to predator odor (Leong and Packard, 2014) or conditioned fear stimuli (Leong et al., 2015; Goode et al., 2016), produce a bias toward and enhancement of DLS habit memory.

The vast majority of research investigating the effects of emotional arousal within the context of multiple memory systems has examined stress exposure or anxiety that is induced during adulthood. Interestingly, extensive evidence indicates that early life stress (ELS) in rodents, usually administered within the first three post-natal weeks, is often associated with memory impairments in adulthood (for reviews, see McCormick et al., 2010; Bolton et al., 2017). For example, early maternal separation or limited bedding and nesting early in life results in reduced adult memory function across a range of tasks, including, for example, object recognition, object location, and spatial learning in the Morris water maze (Lehmann et al., 1999; Huot et al., 2002; Brunson et al., 2005; Aisa et al., 2007; Ivy et al., 2010; Molet et al., 2016). Chronic stress during adolescence also results in adult spatial memory impairments (Isgor et al., 2004; Avital and Richter-Levin, 2005). Importantly, these memory tasks require cognitive memory processes mediated in part by the hippocampus. Indeed, evidence suggests that the effects of ELS on adult cognitive memory may be attributed to deficits in hippocampal structure and function, such as reduced mossy fiber density, dendritic atrophy, and/or impaired long-term potentiation (LTP; Huot et al., 2002; Chen et al., 2004; Brunson et al., 2005). Although several studies have focused on the effects of ELS on hippocampal-dependent memory, there is some evidence that the enhanced use of DLS-dependent memory observed when emotional arousal is induced in adulthood may also be seen following ELS. In a recent study, limited bedding and nesting during post-natal days 2–9 resulted in greater habitual lever pressing in an instrumental learning task (Xu et al., 2022). Similarly, in human subjects, ELS is associated with greater adult use of habit memory strategies in solving instrumental learning tasks (Patterson et al., 2013, 2019; Gordon et al., 2020), and prenatal stress has been similarly associated with increased used of striatal learning strategies in a virtual maze (Schwabe et al., 2012a). Moreover, childhood adversity also correlates with adult scores on a self-report measure of day-to-day habitual tendencies (Creature of Habit Scale; Ersche et al., 2017).

There is accumulating evidence that altered memory system function may be relevant to understanding features of some human psychopathologies. Specifically, maladaptive DLS habit memory might be part of the neural mechanism underlying habit-like behavioral symptoms which manifest in some disorders, such as OCD, PTSD, and drug addiction and relapse (e.g., White, 1996; Everitt and Robbins, 2005; Schwabe et al., 2011; Berner and Marsh, 2014; Gillan and Robbins, 2014; Goodman et al., 2014). It is therefore tempting to speculate that the long-term effects of ELS on memory systems might be part of the mechanism through which ELS might predispose individuals toward developing psychopathologies later in life.

Therefore, the present study investigated how a chronic stress regimen [i.e., repeated corticosterone (CORT) administration] during adolescence influences DLS-dependent habit learning and anxiety in adulthood. In experiment 1, adolescent rats received a single daily injection of either CORT or vehicle over 5 days. Later, when rats reached adulthood, they received training in a habitual response learning version of the water plus-maze task. In this task, animals are trained in a water plus-maze to make the same consistently reinforced body turn response at the maze choice point. Extensive research indicates that acquisition of response learning in the plus-maze task depends on DLS function (for reviews see Packard, 2009; Goodman, 2021). We hypothesized that adolescent CORT administration would enhance acquisition of response learning in adulthood. In experiment 2, separate groups of rats received adolescent CORT administration in a manner similar to experiment 1, while some rats received concurrent administration of the glucocorticoid antagonist mifepristone to determine whether this treatment may block the effects of adolescent CORT on adult habit memory.

## Materials and methods

### Subjects

Adolescent male Long-Evans rats PND 28–35 on arrival were used and were pair-housed with access to food and water *ad libitum* throughout the study. Subjects were housed in a temperature-controlled vivarium with a 12:12 light-dark cycle (lights on 07:30). All procedures were carried out during the light-phase of the cycle. All animal procedures were carried out in accordance with the Texas A&M University Institutional Animal Care and Use Committee (IACUC).

### Apparatus

#### Water plus-maze

**FIGURE 1:**
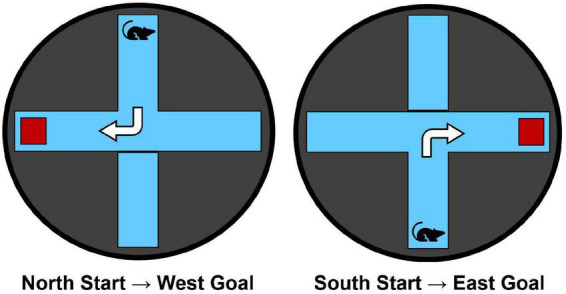
The water maze apparatus and overview of the dorsolateral striatal-dependent response learning task. Starting points from the north and south arms are counterbalanced across trials and training days. Animals are trained to make a consistent body-turn response to reach the hidden escape platform (red square) for 6 trials/day over 7 consecutive training days.

The maze apparatus (Figure 1) was identical to that used in our previous research (e.g., Goodman and Packard, 2014; Goodman et al., 2015). Animals were trained in a black circular water maze (1.83 m in diameter, 0.58 m in height) filled to a water level of 20 cm. A clear Plexiglas plus-maze (43 cm in height, 25 cm in arm-width, and 60 cm in arm length) was inserted into the water maze. The water temperature was maintained at 25°C. A clear Plexiglas escape platform (11 cm × 14 cm) was located 1 cm below the water level in the appropriate maze goal arm.

#### Elevated plus-maze

The EPM consisted of three distinct zones: two opposing closed arms (41.9 cm × 13.6 cm), two opposing open arms (43.2 cm × 14.3 cm), and a center zone at the intersection, all elevated 78.7 cm above the ground. The maze was placed in the center of a novel room (i.e., different from the maze training room), and the room was evenly lit. Exploratory behavior was recorded *via* a webcam placed above the maze and subsequently analyzed *via* manual review.

### Drugs and injection procedures

Animals were handled and weighed daily for 5 days prior to initial drug administration. (CORT; Sigma-Aldrich) was dissolved in a sesame oil (Veh). The dose (5 mg/kg) was selected for its use in prior ELS models (e.g., Veenit et al., 2013) and its ability to mimic a physiological stress response in rats (Stein-Behrens et al., 1994; Venero et al., 1996). Mifepristone (MIFP; Cayman Chemical) was dissolved in a vehicle mixture (mVEH) of 90% physiological saline, 5% DMSO, and 3% Tween 80. In *Experiment 1*, rats received a subcutaneous (s.c.) injection of CORT (5 mg/kg; *n* = 12) or Veh (*n* = 11) once daily for 5 consecutive days. In *Experiment 2*, rats received a s.c. injection of (1) either MIFP (30 mg/kg) or VEH 15-min prior to subsequent administration of (2) either CORT (5 mg/kg) or cVEH. Thus, animals were divided into 4 groups based on treatment conditions: Veh + Veh (*n* = 10), MIFP + Veh (*n* = 8), MIFP + CORT (*n* = 10), and Veh + CORT (*n* = 12).

### Behavioral procedures

All behavioral procedures were conducted once subjects had matured into adulthood (PND 60 +), 3 weeks after the last injection(s). All behavioral procedures and training parameters were held constant across both experiments.

#### Water plus-maze training

Rats were trained in a response learning task within the water plus-maze to reach a hidden escape platform for 6 trials/day over 7 days. Each trial began with placing the rat facing away from the maze center into either the N or S arm, pseudo-randomly counterbalanced across the 6 trials for each day of training. A maximum swim time of 60-s was allocated to reach the escape platform that was always hidden in the right arm relative to the start arm (i.e., N-start = W-goal, S-start = E-goal; Figure 1). Thus, rats were trained to make the same right body turn at the choice point to optimally reach the escape platform. Rats remained on the escape platform for 10-s before the experimenter removed them from the maze. After each trial, rats were placed in an opaque acrylic box for a 30-s inter-trial interval (ITI). Each trial was scored based on the subject’s initial full-body entry into either the correct or incorrect arms. Latency to reach the escape platform was also recorded.

#### Elevated plus-maze

A total of 48-h after the final water plus-maze training session, rats were placed in the EPM center facing an open arm to initiate the session and left to freely traverse the maze for 5-min. The total time spent within the center, open and closed arms as well as the number of entries to each zone were recorded. Time within the open and closed zones was accumulated only when all four paws were within the respective arms’ boundary.

## Results

### Experiment 1

**FIGURE 2:**
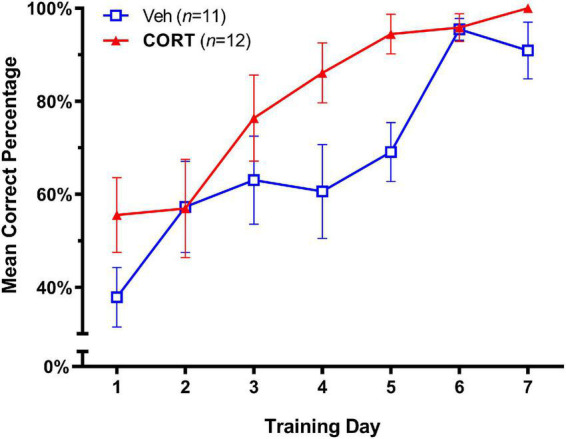
Effect of CORT administration during adolescence on response learning in adulthood (Mean Percent Correct ± SEM). Exogenous CORT administration significantly enhanced task acquisition.

The effects of adolescent administration of CORT on response learning in adulthood are illustrated in Figure 2. A significance level of *p* < 0.05 was employed for all tests. A two-way repeated-measures ANOVA (Group X Day) showed no Group X Day interaction on accuracy scores; *F*_(6,126)_ = 1.473, *p* > 0.05. As expected, both groups showed improved accuracy in task performance over the seven training days, Day, *F*_(3.273,68.73)_ = 17.57, *p* < 0.0001. There was also a strong trend for group differences; Group effect *F*_(1,21)_ = 4.31, *p* = 0.0504, consistent with the hypothesis of enhanced response learning in adulthood in the CORT group. To provide a measure of the magnitude of the treatment effect, we next calculated effect sizes for group differences over days using Cohen’s d (Cohen, 1988), where *d* = 0.50 and *d* = 0.80 are considered medium and large effect sizes, respectively. This analysis revealed large effect sizes for the difference between CORT and vehicle animals that increased across Day 4 (*d* = 0.88) and Day 5 (*d* = 1.39) of training, consistent with an enhancing effect of CORT on task acquisition.

**FIGURE 3:**
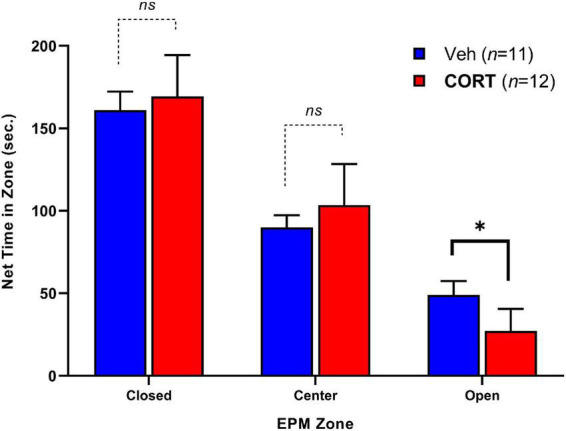
Effect of CORT administration during adolescence on anxiety-like behavior in EPM adulthood. Time spent in the Closed, Center, and Open arms of the apparatus. CORT administration reduced the time spent in the open arms. *p < 0.05.

Elevated plus-maze exploratory behavior between groups was assessed by the time spent in each zone over 5-min (300-s) and is illustrated in Figure 3. Adult rats that had received CORT during adolescence spent less time in the open zones relative to controls, *t*(13.98) = 2.34, *p* < 0.05. No significant differences were observed for time spent in the center, *t*(17.28) = 0.62, *p* > 0.05, or closed arms, *t*(20.88) = 1.32, *p* > 0.05. There were no significant differences in the number of entries into each zone between groups. The EPM findings indicate that chronic adolescent CORT administration can increase anxiety-like behavior in adulthood.

### Experiment 2

**FIGURE 4:**
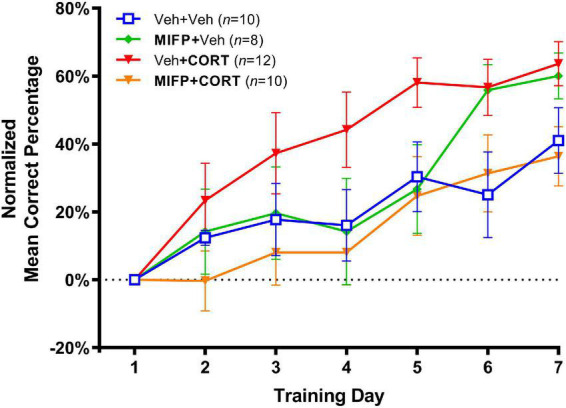
Effects of CORT and mifepristone (MIFP) administration during adolescence on response learning in adulthood (Mean Percent Correct ± SEM). CORT administration enhanced task acquisition and this effect was blocked by concurrent MIFP.

The effects of adolescent administration of CORT on response learning in adulthood, along with the effects of concurrent administration of mifepristone are illustrated in Figure 4. In view of the early training variability inherent with the larger number of groups in experiment 2, the percent correct data was normalized by calculating the difference between percent correct on each training day and percent correct on day one of training. As such, we analyzed the relative rate of increase in habit memory expression in the maze as compared to the first day of training. A two-way repeated-measures ANOVA (Group X Day) revealed a significant group effect, *F*_(3,36)_ = 2.464, *p* < 0.001. The Group X Day interaction was non-significant, *F*_(18,216)_ = 1.428, *p* > 0.05, and a significant effect of Day revealed that all groups improved in task accuracy over the seven training days, *F*_(3.811, 137.2)_ = 27.91, *p* < 0.001.

We next employed Tukey’s *post-hoc* tests and also calculated Cohen’s d for effect size in order to compare group differences in task acquisition. Animal’s receiving Veh + CORT were significantly enhanced in task acquisition relative to Veh + Veh controls, *t*(20) = 3.09, *p* < 0.05; *d* = 1.79. Animals receiving Veh + CORT were also enhanced in task acquisition relative to those administered MIFP + CORT, *t*(20) = 3.37, *p* < 0.01; *d* = 1.95, indicating that the enhancing effect of CORT on response learning was blocked by administration of MIFP. Finally, animals receiving MIFP alone (MIFP + Veh), were not significantly different than the vehicle group (Veh + Veh), *t*(20) = 0.84, n.s.; *d* = 0.40, indicating that MIFP at this dose does not by itself affect task acquisition.

Taken together, these findings indicate animals receiving CORT injections displayed facilitated response learning in adulthood, consistent with the effect of CORT observed in experiment 1. Concurrent injections of the CORT antagonist mifepristone during adolescence blocked the enhancing effect of CORT on response learning, and mifepristone did not affect task acquisition when administered alone.

## Discussion

The present study investigated the effects of adolescent ELS exposure on anxiety and DLS-dependent habit memory in adulthood. Chronic systemic CORT administration during adolescence was used as a model of ELS, based on previous research (e.g., Veenit et al., 2013) and its ability to mimic a physiological stress response in rats (Stein-Behrens et al., 1994; Venero et al., 1996). The present findings indicate that this regimen of CORT administration in adolescence increased anxiety-like behavior during adulthood in an elevated plus-maze. The findings also indicated that chronic CORT treatment during adolescence enhanced acquisition in a DLS-dependent response learning water plus-maze task. Moreover, the effect of adolescent CORT on acquisition was blocked by concurrent administration of glucocorticoid receptor antagonist mifepristone, suggesting the effects of CORT may depend, in part, on glucocorticoid receptor activation.

The findings are largely consistent with extensive prior evidence demonstrating that stress and anxiety may enhance the memory processes of the DLS (for reviews, see Packard and Goodman, 2012; Schwabe, 2013; Packard et al., 2018). Most of this prior research involved the use of stressors and/or anxiogenic drugs administered specifically during adulthood, as opposed to adolescence. Adult administration of several behavioral stressors, such as chronic restraint stress, exposure to predator odor, or anxiogenic drugs enhances the acquisition of habit memory and biases animals toward the use of habit strategies in tasks that may be solved adequately with an alternative strategy (Packard et al., 2018). Consistent with the present findings, CORT administration during adulthood also enhances memory in habitual response learning tasks in rodents (Quirarte et al., 2009; Goodman et al., 2015; Siller-Pérez et al., 2017). In addition, hydrocortisone administration in adult human subjects similarly enhances habit memory in both instrumental learning and virtual navigation tasks (Schwabe et al., 2010, 2012b; Guenzel et al., 2014).

Importantly, the present study adds to emerging evidence that these commonly observed effects of adult stress on DLS habit memory may be mimicked by ELS. Consistent with the present findings, limited bedding, and nesting during post-natal days 2–9 results in greater habitual lever pressing in an instrumental learning task (Xu et al., 2022). Moreover, social isolation during adolescence in mice also enhanced habitual responding to the instrumental task during adulthood (Shan et al., 2022). Our findings are also consistent with accumulating evidence from human research linking ELS to increased use of DLS-dependent habitual responding in adulthood (Patterson et al., 2013, 2019; Gordon et al., 2020). Similar observations have also been made for human prenatal stress, which is associated with enhanced habit memory in adulthood in a virtual-navigation task (Schwabe et al., 2012a). In addition, childhood adversity (as measured by the Childhood Trauma Questionnaire; Bernstein et al., 2003) has been positively correlated with self-report measures of habitual responding in adulthood (measured using the Creature of Habit Scale; Ersche et al., 2017).

While adult administration of glucocorticoids has been associated with enhanced DLS habit memory in prior research, our present findings are the first to our knowledge linking ELS CORT to enhanced habit memory in adulthood. Chronic stimulation of glucocorticoid receptors in adolescence may permanently alter the function of memory systems in the brain favoring habit memory, and this effect may be achieved *via* different mechanisms. Though administered systemically, CORT readily crosses the blood-brain barrier and may bind to high-affinity mineralocorticoid and low-affinity glucocorticoid receptors across the rodent brain (Reul and de Kloet, 1985; de Kloet et al., 1993). Importantly, the DLS may be an important site of action for the effects of adolescent CORT, considering that this brain region richly expresses glucocorticoid receptors; however, this brain region is relatively devoid of mineralocorticoid receptors (Ahima and Harlan, 1990, 1991; Morimoto et al., 1996). As noted previously, the DLS is critically involved in the acquisition, consolidation, and retrieval of habit memory, including the acquisition of habit memory in the specific response learning plus-maze task employed in the present experiments (for review, see Goodman, 2021). It is possible that, in the present study, chronic CORT influenced habit learning directly *via* stimulation of glucocorticoid receptors in the DLS. Indeed, adult administration of CORT directly into the DLS is sufficient to enhance habit memory (Quirarte et al., 2009; Siller-Pérez et al., 2017), and the effects of glucocorticoid administration on DLS memory processes depend on interactions with other neurotransmitter systems, such as endocannabinoid (Siller-Pérez et al., 2019), cholinergic (Sánchez-Resendis et al., 2012), and adrenergic systems (Goodman et al., 2015). The extent to which the effects of adolescent chronic exposure to glucocorticoids observed in the present study may be explained by similar mechanisms may be investigated in future research.

Considering the long-term effects of ELS observed in this study, it is reasonable that our chronic CORT treatment during adolescence resulted in long-term functional and/or structural changes in one or more memory systems of the brain to produce the observed effects on habit memory. This explanation would be consistent with recent evidence showing that behavioral stressors during childhood/adolescence may enhance habit memory during adulthood *via* altering structure and function of the dorsal striatum. Limited bedding and nesting during childhood in mice boosts habitual responding later in life, and this enhancement was associated with the increased post-synaptic density of neurons in the DLS (Xu et al., 2022). Moreover, ELS may be associated with suppression of the striatonigral direct pathway of the dorsomedial striatum (DMS; Shan et al., 2022), a brain region implicated in goal-directed action-outcome learning as opposed to DLS-mediated habitual stimulus-response learning (Yin and Knowlton, 2006; Dezfouli and Balleine, 2012). Importantly, chemogenetic activation of direct pathway DMS neurons erases the enhancing effect of ELS on habit memory and shifts animals away from DLS-habitual responding and toward goal-directed responding (Shan et al., 2022). Future research is required to determine whether the ELS treatment employed in the present experiments (i.e., chronic CORT treatment) enhances habit memory *via* similar structural and functional changes in the dorsal striatum. Considering that CORT was administered systemically, it should be noted that additional brain regions outside the dorsal striatum may also be involved.

Chronic administration of CORT (similar to behavioral stressors) may have a global impact on the brain, affecting changes in multiple brain areas, including those supporting learning and memory. In addition to structural and functional changes in the dorsal striatum, it is possible that ELS may enhance habit memory indirectly *via* changes to the hippocampus. While the hippocampus does not mediate habit memory, evidence suggests that the hippocampus may interact with the DLS, and in some learning situations, these memory systems compete for control of behavioral output (for reviews, see Poldrack and Packard, 2003; Packard and Goodman, 2013; Goodman, 2021). For instance, extensive evidence suggests that hippocampal impairments (e.g., reversible lesion) can lead to *enhancement* of DLS-dependent habit memory (Packard et al., 1989), and this effect is mimicked by stress and anxiety-induced impairments to the hippocampus (e.g., Kim et al., 2001; Wingard and Packard, 2008). A competitive interaction between the hippocampus and DLS may be especially relevant to understanding the effects of ELS on habit memory, considering evidence that ELS also impairs the structure and function of the hippocampus. For example, ELS is associated with adult reduction of mossy fiber density, dendritic atrophy, and impaired LTP in the hippocampus (Huot et al., 2002; Chen et al., 2004; Brunson et al., 2005) as well as impairments in hippocampus-dependent declarative/spatial memory (Lehmann et al., 1999; Huot et al., 2002; Isgor et al., 2004; Avital and Richter-Levin, 2005; Brunson et al., 2005; Aisa et al., 2007; Ivy et al., 2010; Molet et al., 2016). Importantly, when stress is administered during adulthood, stress-induced impairments in hippocampal LTP and declarative memory are also associated with enhanced habit memory (e.g., Kim et al., 2001). Thus, ELS-induced hippocampal impairment may be part of the mechanism through which ELS enhances habit memory.

Notably, a recent study suggested that ELS-induced enhancement of DLS and impairment of the hippocampus may both be associated with habit memory enhancement in adulthood (Xu et al., 2022). In this study, ELS increased post-synaptic density and the number of thin and mushroom-shaped spines in the DLS, whereas opposite effects were found in the CA1 region of the hippocampal neurons (Xu et al., 2022). These structural changes were correlated with greater reliance on habitual responding in an instrumental learning task and impaired spatial memory in a novel object location task (Xu et al., 2022). Thus, consistent with a competitive interaction between memory systems (Poldrack and Packard, 2003), it is possible that ELS-induced structural changes to the hippocampus and DLS may similarly account for, in part, the enhancing effects of ELS on habit memory observed in the present experiments. Further research will be required to investigate these and other potential mechanisms.

Research on ELS is highly relevant to understanding how childhood or adolescent stressful life events, trauma, and abuse can result in the development of psychiatric disorders later in life (Carr et al., 2013; Syed and Nemeroff, 2017). In light of the present findings, it is possible that ELS may lead to heightened DLS habit memory in adult human psychiatric patients. Multiple investigators have suggested that stress and anxiety may be, in part, associated with the development and expression of habit-like behavioral symptoms observed in multiple disorders, such as obsessive-compulsive disorder, PTSD, and drug addiction, among other disorders (White, 1996; Everitt and Robbins, 2005; Schwabe et al., 2011; Goodman et al., 2012, 2014; Berner and Marsh, 2014; Gillan and Robbins, 2014; Goodman and Packard, 2016). ELS has been associated with the development of similar disorders in adulthood (e.g., addiction and PTSD, and other anxiety disorders; Enoch, 2011; Nugent et al., 2011). Therefore, ELS-induced changes to memory systems that lead to enhanced habit memory in adulthood may partially account for the habit-like symptoms observed in these adult psychiatric disorders (e.g., avoidance symptoms in PTSD, drug-seeking and relapse, compulsive behaviors, repetitive and stereotyped behaviors, etc.). It is important to emphasize that ELS has a dramatic impact on multiple brain areas and circuits that may be related to adult psychopathology (Gutman and Nemeroff, 2002; Heim and Nemeroff, 2002; Bremner and Vaccarino, 2015; Agorastos et al., 2019; Kirsch et al., 2020), and enhanced DLS habit memory represents only one of the many neurobehavioral outcomes of ELS. Nevertheless, considering how ELS-induced enhancements of habit memory could critically explain some of the hallmark features of adult human psychopathology, this effect and its underlying mechanisms warrant further investigation in future research.

## Data availability statement

The original contributions presented in this study are included in the article/supplementary material, further inquiries can be directed to the corresponding author.

## Ethics statement

The animal study was reviewed and approved by the IACUC Texas A&M University.

## Author contributions

All authors listed have made a substantial, direct, and intellectual contribution to the work, and approved it for publication.
